# Controlled Release of Radioactive Water from the Fukushima Daiichi Nuclear Power Plant: Should We Be Concerned?

**DOI:** 10.1021/acs.est.3c08702

**Published:** 2024-03-07

**Authors:** Maria F. Ferreira, Andrew Turner, Awadhesh N. Jha

**Affiliations:** † School of Biological and Marine Sciences, University of Plymouth, Drake Circus, Plymouth PL4 8AA, U.K.; ‡ School of Geography, Earth and Environmental Sciences, University of Plymouth, Drake Circus, Plymouth PL4 8AA, U.K.

**Keywords:** radioactive water, tritium (^3^H), human health, environmental impact, Fukushima Daiichi

Following the Fukushima Daiichi nuclear disaster in 2011, the decision to release more than 1 million tons of radioactive water into the ocean by the Japanese government, with approval from the IAEA, has divided public and scientific opinion. The discharge began on August 24, 2023, with the premise that, after removal of long-lived radionuclides (i.e., ^137^Cs and ^90^Sr), tritium (^3^H), the primary remaining radionuclide as tritiated water (HTO), will be sufficiently and safely diluted over a 30-year period.^1^ Concerns, however, relate to (a) the safety of seafood and its consumers and (b) potential long-term consequences on human and environmental health.^2,3^

## Behavior of Tritium in the Environment

**Figure 1:**
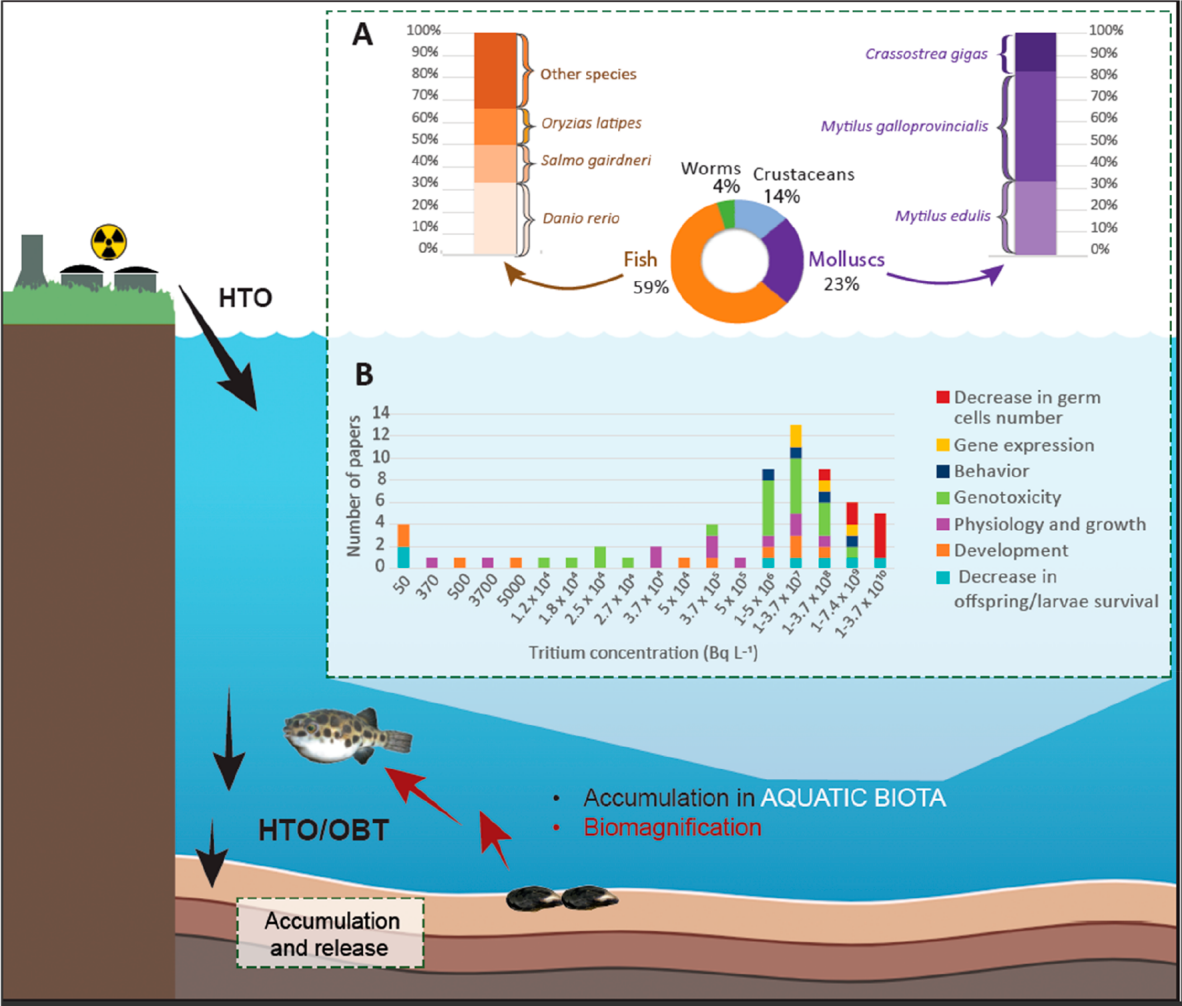
Assessment of the behavior of tritium (^3^H) in the aquatic environment and impacts on biota. (A) Animal groups covered and (B) biological effects reported according to data compiled by Ferreira et al.^3^ Abbreviations: HTO, tritiated water; OBT, organically-bound tritium.

It is known that ^3^H (half-life of 12.6 years) in its inorganic form (i.e., HTO) quickly integrates into biological systems and can consequently associate with organic molecules [as organically bound-tritium (OBT)].^4,5^ The environmental persistence of OBT, including in sediments and soils, raises concerns about its potential transfer to the water cycle and its biomagnification through the food web.^3^ For example, in the Severn Estuary (U.K.), it has been suggested that bioaccumulation of anthropogenic OBT by benthic organisms and demersal fish largely occurs via a pathway of conversion of dissolved OBT into particulate OBT (through bacterial uptake and physicochemical processes) and subsequent transfer up the food web by sediment-dwelling microbes and meiofauna.^6^ Moreover, the pattern of distribution of OBT among different tissues in organisms depends on not only the chemical or biochemical characteristics of each tritiated compound but also the metabolic activities of different tissues.^5,7^ Data on HTO and OBT distribution, behavior, and potential effects are available for only a few taxonomic groups and are heavily biased toward laboratory species (Figure 1). This contributes to considerable uncertainty in environmental risk assessments (ERA) for the radionuclide.^3^

## Radiation Dose Estimations and Effects on Humans and Non-human Biota

It has been suggested that doses to biota arising from exposure to controlled but continuously or intermittently discharged HTO will be far below the acceptable radiation threshold for human consumption.^1^ This assumption is largely based on external dose estimations, with internal doses and bioaccumulation potential not factored in.

Although it is a β-emitter, the relative biological effectiveness (RBE) of ^3^H in tissues is contested. Laboratory studies have shown that ^3^H could be as potent as high-energy γ or X-ray radiation for the induction of chromosomal damage in fish.^8^ A precautionary approach has been recommended while dealing with internal emitters, given that ionizing radiation could induce many other novel effects that have thus far not been considered in risk assessments. These include genomic instability (non-clonal damage), bystander effects, minisatellite mutations, and epigenetic changes.^9^ With regard to human health, higher incidences of chromosomal aberrations have been reported in workers exposed to ^3^H compared to unexposed individuals,^10^ while higher incidences of childhood leukemia around nuclear power plants, attributed to ^3^H exposures, have been hotly debated in the scientific community.^11^

Compared with human health, dose estimation for natural biota, which exhibit different geometric shapes and sizes, is challenging and not well-defined.^3,12^ The external dose estimation based on energy levels of different qualities of radiation^1^ cannot be considered realistic and safe for either non-human biota or humans exposed through different routes (e.g., inhalation, food, water, and skin). In this context, chronic exposure to HTO can induce DNA damage at different life stages of marine species.^3^ In particular, an increased level of chromosomal damage was observed in blue mussels following a 7-day exposure to OBT, at a dose rate of 4.9 μGy h^–1^;^5^ this is lower than the suggested generic (all species) “no effect” dose rate limit of 10 μGy h^–1^.^13^

## Radiation Accidents and *In Situ* Studies

The Fukushima Daiichi and Chernobyl accidents are often directly and erroneously compared without taking into consideration their different environmental scenarios.^1^ The Fukushima Daiichi nuclear power plants are directly adjacent to the open ocean, whereas Chernobyl is 500–600 km from the semi-enclosed Baltic and Black Seas. While Fukushima Daiichi represents the largest accidental release of radionuclides to the ocean in terms of measured radionuclide concentrations,^14^ available reports suggest that HTO was not released following the Chernobyl accident. In addition, Fukushima-derived radionuclides have been shown to be transported long distances by marine fish,^15^ making the accident a global concern.

Biological studies carried out in the Chernobyl region are controversial, and it is by no means conclusive that biota are resilient.^16^ For example, histological impacts on fish gonads and altered expression of genes have been demonstrated 30 years after the accident.^17^ Significantly, sublethal effects of ^3^H in fish inhabiting rivers near a Canadian nuclear site include DNA damage in blood and gametes; this could affect the physiological and reproductive fitness and, ultimately, the genetic diversity of a population.^18^ To the best of our knowledge, no study has addressed the impacts of HTO at the ecosystem level, and studies in areas with above-background levels of radiation are scarce.

## Concluding Remarks

Accumulated radioactive water cannot be stored indefinitely at Fukushima Daiichi due to the ongoing risk of earthquakes and tsunamis in the region. However, the environmental behavior of different forms of ^3^H, including the mechanisms and rates of OBT formation, needs to be studied further to better define and understand its potential long-term impacts. In addition to regular monitoring, future studies should include more realistic environmental scenarios in the presence of multiple, emerging stressors, such as hypoxia, higher temperatures,^19^ and microplastics.^20^ The development of modeling approaches to accurately estimate doses for different radionuclides in biota is also vital. Given the quantities of HTO discharged globally,^1^ a fundamental goal should be the minimization of its production and discharge.
